# Influence of Plasma-Isolated Anthocyanins and Their Metabolites on Cancer Cell Migration (HT-29 and Caco-2) In Vitro: Results of the ATTACH Study

**DOI:** 10.3390/antiox11071341

**Published:** 2022-07-08

**Authors:** Inken Behrendt, Isabella Röder, Frank Will, Hamza Mostafa, Raúl Gonzalez-Dominguez, Tomás Meroño, Cristina Andres-Lacueva, Mathias Fasshauer, Silvia Rudloff, Sabine Kuntz

**Affiliations:** 1 Department of Nutritional Science, Human Nutrition, Justus-Liebig-University, 35390 Giessen, Germany; mathias.fasshauer@ernaehrung.uni-giessen.de (M.F.); sabine.kuntz@nutr.jlug.de (S.K.); 2 Department of Beverage Research, Hochschule Geisenheim University, 65366 Geisenheim, Germany; isabella.roeder@googlemail.com (I.R.); frank.will@hs-gm.de (F.W.); 3 Biomarkers and Nutrimetabolomics Laboratory, Department of Nutrition, Food Sciences and Gastronomy, Food Innovation Network (XIA), Nutrition and Food Safety Research Institute (INSA), Facultat de Farmàcia i Ciències de l’Alimentació, Universitat de Barcelona (UB), 08028 Barcelona, Spain; hamza_mohamedamin@ub.edu (H.M.); raul.gonzalez@ub.edu (R.G.-D.); tomasmerono@ub.edu (T.M.); candres@ub.edu (C.A.-L.); 4 Centro de Investigación Biomédica en Red de Fragilidad y Envejecimiento Saludable (CIBERFES), Instituto de Salud Carlos III, 28029 Madrid, Spain; 5 Department of Nutritional Science and Department of Pediatrics, Justus-Liebig-University, 35392 Giessen, Germany; silvia.rudloff@ernaehrung.uni-giessen.de

**Keywords:** anthocyanins, migration, intervention study, colon cancer, 5-fluorouracil, grapes, bilberry, antioxidant capacity, juice

## Abstract

Cancer mortality is mainly due to metastasis. Therefore, searching for new therapeutic agents suppressing cancer cell migration is crucial. Data from human studies regarding effects of anthocyanins on cancer progression, however, are scarce and it is unclear whether physiological concentrations of anthocyanins and their metabolites reduce cancer cell migration in vivo. In addition, interactions with chemotherapeutics like 5-fluorouracil (5-FU) are largely unknown. Thus, we combined a placebo-controlled, double-blinded, cross-over study with in vitro migration studies of colon cancer cell lines to examine the anti-migratory effects of plasma-isolated anthocyanins and their metabolites (PAM). Healthy volunteers (*n* = 35) daily consumed 0.33 L of an anthocyanin-rich grape/bilberry juice and an anthocyanin-depleted placebo juice for 28 days. PAM were isolated before and after intervention by solid-phase extraction. HT-29 and Caco-2 cells were incubated with PAM in a Boyden chamber. Migration of HT-29 cells was significantly inhibited by PAM from juice but not from placebo. In contrast, Caco-2 migration was not affected. Co-incubation with 5-FU and pooled PAM from volunteers (*n* = 10), which most effectively inhibited HT-29 migration, further reduced HT-29 migration in comparison to 5-FU alone. Therefore, PAM at physiological concentrations impairs colon cancer cell migration and may support the effectiveness of chemotherapeutics.

## 1. Introduction

Colorectal cancer (CRC) is one of the most common cancer types worldwide. In 2020, more than one million patients were newly diagnosed and ~600,000 CRC deaths occurred [1]. However, CRC incidence shows distinct geographic variations, with higher incidence rates in industrialized countries revealing Western lifestyle, and particularly Western diet, as major modifiable risk factors [2,3]. Although the 5-year relative survival rate for CRC patients with localized disease is about 90% [4], metastasis is associated with poor outcomes. In CRC patients with metastasis, the 5-year relative survival rate dramatically declines to 14% [4]. Therefore, CRC is the second leading cause of cancer death globally [1] and searching for new therapeutic agents to impair tumor progression and metastasis is critical.

Carcinogenesis and metastasis are associated with oxidative stress that is characterized by excessive levels of reactive oxygen species (ROS) [5]. ROS are highly reactive and capable of damaging macromolecules such as DNA, proteins, and lipids, as well as cellular structures, thus promoting malignant transformation [5,6]. In addition, mitochondrial dysfunction and high ROS levels increment the migratory and invasive potential of several cancer cell lines [7,8,9]. Therefore, antioxidants are hypothesized as chemopreventive and chemotherapeutic agents [6]. In recent years, several plant-derived phytochemicals, particularly polyphenols, have attracted remarkable attention for their potential to prevent tumor initiation, promotion, and progression, due to their low cost, low toxicity, and the undesirable adverse side effects of chemotherapeutical drugs [10]. Anthocyanins, a subgroup of flavonoids, belong to the most prevalent group of polyphenols in fruits [11] and are responsible for their orange to bluish-red color [12,13,14]. Pelargonidin, cyanidin, delphinidin, peonidin, petunidin, and malvidin are the predominant dietary anthocyanidins. These six anthocyanidins account for more than 90% of all yet identified anthocyanins [15]. However, anthocyanins in fruits are primarily present as glycosides or acylated glycosides. Berries such as blueberries, blackberries, raspberries, cranberries, and grapes show anthocyanin contents between 21 and 390 mg per 100 g fresh weight [16] with peonidin and cyanidin being the major anthocyanins in grapes and berries, respectively [17]. It is well known, that due to their structural polyphenolic characteristics, anthocyanins exhibit a high antioxidative potential, e.g., because of their capability for donating electrons [18], scavenging ROS [19,20], preventing ROS-induced oxidative damage or influencing antioxidative enzyme expression [21,22]. In epidemiological studies, total dietary anthocyanin intake is inversely associated with CRC [18,23,24]. In addition, numerous in vitro and in vivo studies reveal that anthocyanins are able to decelerate CRC promotion and progression by several mechanisms such as triggering cell cycle arrest and apoptosis or inhibiting proliferation and invasion by different signaling pathways [10,17], whereas studies regarding the anti-migratory effects of anthocyanins on colon cancer cells are scarce [25,26,27]. Nonetheless, it is also known that the bioavailability of anthocyanins is relatively low and native anthocyanins are only detectable at very low concentrations in the systemic circulation [28,29]. Anthocyanins which are not absorbed in the duodenum reach the lower gastrointestinal tract where they are metabolized by the gut microbiota. Recently, it has been shown that microbially metabolized anthocyanin compounds account for the majority of absorbed berry phenols [30]. Moreover, these anthocyanin metabolites also show bioactive properties and seem to be more effective than native anthocyanins to reduce Caco-2 cell proliferation [17]. In this context, we have recently shown for the first time that physiological concentrations of anthocyanins and their metabolites isolated from the plasma of healthy volunteers after a single dose of an anthocyanin-rich juice were able to reduce tumor cell migration of the pancreatic cancer cell line PANC-1 in vitro [31]. This was accompanied by a significant reduction in ROS levels and decreased matrix metalloproteinases (MMP-2 and MMP-9), as well as NF-κB, mRNA expression [31]. Despite the common concept of antioxidants as tumor suppressors, recent evidence indicates that antioxidants may also act as tumor promotors, especially in metastasis [6]. Therefore, the role of ROS and antioxidants such as anthocyanins during metastasis are still not fully understood [32]. Similarly, the prognostic importance of enzymatic and non-enzymatic biomarkers of oxidative stress in colorectal cancer progression and metastasis is still unclear. In CRC patients biomarkers of oxidative stress are significantly increased, whereas the total antioxidant capacity (TAC) is significantly lower compared with healthy controls [5,33]. Thus, CRC patients may be more vulnerable to oxidative stress and administering of anthocyanins might improve the antioxidant capacity, overall health, and the outcome for CRC patients.

Therefore, the primary outcome of the present study was to determine whether physiological concentrations of anthocyanins and their metabolites, isolated from plasma of healthy volunteers after long-term consumption of an anthocyanin-rich grape/bilberry juice, impair the migratory potential of two colon cancer cell lines, HT-29 and Caco-2, in vitro. Although both cell lines were isolated from adenocarcinoma and exhibit epithelial phenotypes [34,35,36], HT-29 and Caco-2 cells were chosen due to their varying grade of differentiation [37] and their different migratory, as well as metastatic, potential [36]. We further aimed to investigate whether possible anti-migratory effects were associated with alterations of antioxidant status parameters in the plasma or urine of the volunteers (secondary outcome).

## 2. Materials and Methods

### 2.1. Preparation and Characterization of the Anthocyanin-Rich Juice and the Anthocyanin-Depleted Placebo

The anthocyanin-rich juice and the anthocyanin-depleted placebo were produced at the Hochschule Geisenheim University (Department of Beverage Research, Geisenheim, Germany) and were similar to the juices for the ANTHONIA study with minor modifications [21,38]. Briefly, juices were made from 80% red grape juice (grape variety *Accent*) and 20% bilberry juice (Heidelbeersaft blank BIO (Bayernwald KG, Hengersberg, Germany). Grapes were extracted in a press and the resulting juice was separated, blended with the bilberry juice, pasteurized, and hot-filled into 0.33 L brown glass bottles. Placebo juice was obtained by passing the juice through SP70 Sepabeads^®^ absorber resin (Resindion S.r.l., Binasco, Italy). Both juices were analyzed directly after membrane filtration (0.45 µm) for basic analytical parameters such as total phenolics, concentrations of anthocyanins, and TEAC (Trolox equivalent antioxidative capacity) as described elsewhere [39]. Anthocyanins were analyzed by LC–MS as previously described [21]. Quantitation was carried out in duplicate using peak areas detected at 520 nm and based on external calibration via the reference substance cyanidin-3-O-glucoside (0.1–100 mg/L; linearity of calibration, r^2^ = 0.9999). For cyanidin-3-O-glucoside, the limit of detection was 0.01 mg/L and the limit of quantitation was 0.04 mg/L.

### 2.2. Study Design and Study Subjects

The randomized, placebo-controlled, double blinded, cross-over ATTACH study (Anthocyanins Target Tumor cell Adhesion—Cancer vs. Endothelial Cell (HUVEC) Interactions study) was carried out at the Department of Nutritional Science, Justus Liebig University, Giessen (Germany) between April and August 2019. Sample size was calculated based on the results of our previous published migration study [31] with a β- and α-error of 0.8 and 0.05 and a drop-out rate of 20%. Calculations were made with Stata Version 15.1 (StataCorp LLC, College Station, TX, USA) from ASKNET solutions AG (Karlsruhe, Germany). In total, 45 healthy students from the Justus Liebig University, Giessen were recruited and due to exclusion criteria (drugs and antibiotics in the last 3 months before the study, vitamin and mineral supplementation, as well as intestinal or cardiovascular diseases) 2 were excluded. After randomization 8 declined to participate due to non-compliance with the nutritional recommendations. From the 35 volunteers (female *n* = 27 and male *n* = 8), 13 were omnivores, 8 vegetarians, 2 vegans and 1 pescetarian with a mean ± SD age of 24.4 ± 2.3 years (range: 19–29 years), an initial body weight of 64.3 ± 17.9 kg (range: 49–93 kg), and a BMI of 21.7 ± 2.6 kg/m^2^ (range: 18.4–27.6 kg/m^2^). Baseline characteristics did not change during the total study period. Of the 35 subjects, 34 collected all samples (blood and urine), whereas one subject had an incomplete urine sample collection.

**Figure 1:**
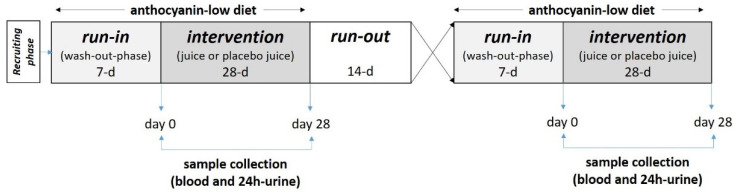
Study design of the ATTACH study. Participants consume the anthocyanin-rich juice and the anthocyanin-depleted placebo. Before (day 0) and after each intervention (day 28), blood and 24 h-urine samples were collected and processed for biochemical analyses (*n* = 35). d, day.

Volunteers received the beverages, denoted as “one” or “two”, weekly at the Department of Nutritional Science. The beverages were filled in brown bottles to ensure blinding and distributed weekly to the volunteers by the lab personal. Participants were instructed to daily consume 0.33 L of the anthocyanin-rich juice or the anthocyanin-depleted placebo for 28 days. They were instructed to keep the juices cool and to avoid their exposure to direct light. Thus, after a 7-day wash-out period, a 28-day intervention period followed, and the first phase was completed by a 14-day run-out period. After the run-out phase, the next phase started with the next beverage (Figure 1).

As phenolic compounds including anthocyanins are present in many foodstuffs and beverages, participants were explicitly counseled prior to the start of the study to follow a low phenolic/anthocyanin diet during the run-in and intervention period to avoid possible effects of other phenolics, especially anthocyanins, from the diet. Similar to our ANTHONIA study, the volunteers received a list of categorizing foodstuffs including beverages into “quantitatively limited” and “not being allowed” with slight modifications [21]. Foodstuffs were categorized according to their anthocyanin content based on data using the USDA Database for the Flavonoid Content of Selected Foods (release 3.3 (2018); http://www.ars.usda.gov/, accessed on 1 March 2019) or a database on polyphenol contents in food (Polyphenol-Explorer, release 3.0; http://www.phenol-explorer.eu/compounds, accessed on 1 March 2019) (Table S1).

Participants were also instructed to record their dietary intake over 3 days during each intervention period in order to estimate their daily energy and nutrient intakes. Dietary records were analyzed using the DGE-PC Professional software, version 1.10.0.0 (Table S2). Participants were instructed to maintain their usual physical activity.

The study protocol was approved by the local ethics committee (registration number 13/10) and according to the guidelines laid down in the Declaration of Helsinki. It is registered at DRKS (Deutsche Register Klinischer Studien) with the registration number DRKS00014767. Written informed consent was obtained from all included participants and data collection was conducted by the Department of Nutritional Science (Giessen, Germany).

### 2.3. Collection of Plasma and Urine Samples

Sample collection occurred before and after the two intervention periods. After an initial 7-day run-in period, participants were instructed to collect their 24 h-urine and blood was drawn by venipuncture into tubes with EDTA as anticoagulant (Sarstedt & Co., Nuembrecht, Germany). Plasma was separated immediately by centrifugation (1200× *g* for 15 min; 4 °C). The supernatant was divided into aliquots and stored at −80 °C until assayed (day 0). Volunteers again collected their 24 h-urine at the end of the intervention and consumed the juice after an overnight fast together with breakfast on the last day of the intervention period. Plasma samples were collected 6 h after juice ingestion (day 28).

### 2.4. Isolation of Plasma Anthocyanins and Their Metebaolites by Solid Phase Extraction

Plasma extraction of anthocyanins and their metabolites was based on the method described recently [40]. Briefly, each aliquot (1 mL of plasma acidified with 30 µL of 50% aqueous formic acid (Lgc Promochem, Wesel, Germany)) was loaded onto an Oasis-HLB (1 mL/30 mg) SPE cartridge (Waters, Inc., Eschborn, Germany), preconditioned with 1 mL of methanol (Thermo Fischer Scientific, Langenselbold, Germany) and 1% formic acid, followed by 1 mL of acidified water (1% formic acid). The cartridge was then washed with 1 mL of acidified water, after which anthocyanins and their metabolites were eluted with 1 mL of acidified methanol. Afterwards, eluates were dried under N_2_ for approximately 3 h [40]. For cell migration studies with HT-29 and Caco-2 cells, dried anthocyanins isolated from plasma as well as their metabolites (PAM) were resolved in the same volume of culture media (1 mL; pH 7.2) as the original plasma volume (1 mL).

### 2.5. Cell Culture

The two human colon carcinoma cell lines HT-29 (HTB-38) and Caco-2 (HTB-37) were purchased from the American Type Culture Collection (ATCC) (San Diego, CA, USA). HT-29 is a cell line with epithelial morphology that was isolated in 1964 from a primary tumor obtained from a 44-year-old female patient with colorectal cancer by J. Fogh [34]. HT-29 cells were grown in RPMI 1640 GlutaMax (Invitrogen GmbH, Darmstadt, Germany) supplemented with 1 mmol/L sodium pyruvate (Invitrogen GmbH, Darmstadt, Germany) and 10% fetal calf serum (FCS) (Invitrogen GmbH, Darmstadt, Germany). Caco-2 are epithelial cells isolated from colon tissue derived from a 72-year-old male with colorectal adenocarcinoma and has been widely used as a model of the intestinal epithelial barrier [35]. Cells were grown in DMEM (Invitrogen GmbH, Darmstadt, Germany) supplemented with 5 mM L-glutamine (Invitrogen GmbH, Darmstadt, Germany), 1 mmol/L sodium pyruvate and 10% FCS. Thus, both cell lines were isolated from colon adenocarcinomas and were most widely used for in vitro studies to compare their tumorigenicity genotype [41,42,43]. Both cell lines were sub-cultured twice a week, incubated at 37 °C in a humidified 5% (*v*/*v*) CO_2_ atmosphere and used between passages 10 to 35. Culture medium was changed every two days.

### 2.6. Cell Migration of Colon Cancer Cells In Vitro

Tumor cell migration was assessed in a Boyden chamber with the use of the CytoSelect 24-well Cell Migration Assay (CellBiolabs, San Diego, CA, USA) according to earlier studies [31]. The feeder trays were coated with 100 µL of 10 µg/mL collagen (Merck GmbH, Darmstadt, Germany) and aspirated until dryness. Onto the upper side of the 24-well feeder chamber (diameter of the chamber 6.5 mm; pore size 8 µm), cells at a density of 1 × 10^5^/mL were seeded in DMEM or RPMI 1640 GlutaMax containing 1% FCS and diluted PAM (day 0 vs. day 28), whereas DMEM or RPMI 1640 GlutaMax supplemented with 10% FCS were added to the lower chamber [41]. Cells were incubated in the feeder tray for 36 h at 37 °C. Then cells on the lower side were detached from the membrane using a cell detachment solution and afterwards lysed with a fluorescent-dye-containing buffer. The extent of migration was assessed by the intensity of the fluorescence signal, which was measured with a Synergy H1 microplate fluorescence reader (Biotek, Karlsruhe, Germany). The number of migrated cells was determined according to a calibration curve (500 to 15,000 cells). Results are expressed as median with interquartile range from *n* = 35 or *n* = 34 volunteers. A 100 mM stock of 5-FU (Sigma-Aldrich Chemie GmbH, Taufkirchen, Germany) was prepared in absolute DMSO (Merck GmbH, Darmstadt, Germany) and stored at −20 °C. The concentration of DMSO was less than 1% of drug treatment. For treatment, 5-FU was diluted in culture media and added to cultures to give the desired final concentration.

### 2.7. Assessment of Cell Viability by Flow Cytometry

Viability of cells was determined by flow cytometry with the Guava^®^ ViaCount^TM^ reagent (Luminex, MV’s, Hertogenbach, Netherlands). Therefore, HT-29 cells were seeded at a density of 1 × 10^5^ cells/mL in 24-well plates in complete medium with or without PAM from the anthocyanin-rich juice, which were isolated before or after the 28-day intervention as well as with different concentrations of 5-FU. After 36 h incubation, cells were washed twice with PBS (Invitrogen GmbH, Darmstadt, Germany) and trypsinized with TrypLE Express (Invitrogen GmbH, Darmstadt, Germany). Cell viability and cytotoxicity were measured according to the manufacturer’s instructions using a Guava^®^ Muse^®^ Cell Analzyer (Luminex, MV’ss, Hertogenbach, Netherlands).

### 2.8. Oxidative Biomarkers in Plasma and Urine Samples

Oxidative biomarkers in plasma and urine samples were measured before (day 0) and after each intervention period (day 28). Enzyme activities of superoxide dismutase (SOD), catalase (CAT), and glutathione peroxidase (GPx) were determined by colorimetric methods according to the manufacturer’s instructions (Cayman Chemical Company, Ann Arbor, MI, USA) in a Synergy H1 microplate fluorescence reader (Biotek, Karlsruhe, Germany) as described previously [21]. The amount of water and lipid soluble antioxidants in plasma was assessed using the ABTS (2,2-azino-di-3-ethylbenzthiazoline sulphonate) assay (Cayman Chemical Company, Ann Arbor, MI, USA). Generation of ABTS^+^ from ABTS by metmyoglobin is inhibited by antioxidants and yields a reduction in absorbance at 405 nm. The capacity of antioxidants to reduce ABTS^+^ generation was compared with that of Trolox and quantified as Trolox equivalents (mmol/L TEAC) [21]. All analyses were performed in duplicate and intra-assay coefficients of the assays were <12%. The total phenolic content (TPC) in urine samples was determined after solid-phase extraction (SPE) (Oasis^®^ MAX 96-well plate cartridges) using a rapid Folin–Ciocalteu method described earlier [44,45]. Briefly, in a thermo microtiter 96-well plate, 170 μL of Milli-Q water, 15 μL of the urine extract, 12 μL of the Folin–Ciocalteu reagent (Sigma-Aldrich Chemie GmbH, Taufkirchen, Germany) and 30 μL of 20% sodium carbonate were mixed. After 1 h of incubation at room temperature in the dark, the absorbance was measured at 750 nm using an UV/VIS Thermo Multiskan Spectrum spectrophotometer (Vantaa, Finland). Gallic acid (Sigma-Aldrich Chemie GmbH, Taufkirchen, Germany) was used as a standard and quantified as gallic acid equivalents (mg/L). All samples were processed in triplicate and the coefficient of variation (CV) was <10%. Results were multiplied by the urine output and expressed as gallic acid equivalents (GAE) in mg/24 h

### 2.9. Statistical Analyses

Data from the volunteers who completed all phases of the study (*n* = 35; *n* = 34) were analyzed. The outcome measures were prospectively designated as the differences in migration of HT-29 and Caco-2 cells in vitro (primary outcome) and antioxidative parameters (secondary outcome) of placebo and juice treatment before (day 0) and after (day 28) intervention. Before-treatment versus after-treatment data within groups were analyzed using a repeated measures one-way ANOVA, with Šídák’s post hoc test. A mixed model with multiple comparison test (Šídák’s) were used for data sets with missing values. The normality of continuous variables was assessed using Kolmogorov–Smirnov normality test. Asterisks are used in the figures to denote *p* values < 0.05, which were considered significant. Data were expressed as mean ± SD or as median with interquartile range (25th–75th percentile). Correlation analyses were evaluated using Spearman correlation (r) with differences between values from day 28 and day 0 after anthocyanin-rich juice intake (e.g., Δ values = values J_28-days—values J_0-days). GraphPad Prism 9 Version 9.3.1. from ASKNET solutions AG (Karlsruhe, Germany) was used for data analyses.

## 3. Results

### 3.1. Composition of the Anthocyanin-Rich Juice and the Anthocyanin-Depleted Placebo

In this cross-over, placebo-controlled intervention study, an anthocyanin-rich grape/bilberry juice was given to healthy male and female volunteers for 28 days in order to investigate the influence of PAM on cancer cell migration in vitro. To eliminate possible effects of other juice compounds, an anthocyanin-depleted placebo juice was also applied. The anthocyanin-rich juice exhibited an especially high antioxidant activity corresponding to a higher TEAC value (27 ± 1.7 mmol/L), as well as higher concentrations of total phenolics (2622 ± 56 mg/L catechin equivalents) and anthocyanins (942 ± 10 mg/L cyanidin-3-O-equivalents) in comparison with the anthocyanin-depleted placebo (TEAC: 1.0 ± 0.04 mmol/L; total phenolics: 115 ± 5.0 mg/L catechin equivalents; anthocyanins: 6.3 ± 0.5 mg/L cyanidin-3-O-glucoside equivalents). In comparison with commercially available grape (TEAC: 10–12 mmol/L) and blueberry juices (TEAC: 13–17 mmol/L) the TEAC value of our juice was approximately 2–3-fold higher [46,47,48]. Regarding anthocyanins, which were the main phenolics in both beverages, the applied beverages considerably differed in their profile and contents (Table 1). Due to the high grape content, peonidin-3,5-O-diglucoside and malvidin-3,5-O-diglucoside were the most abundant anthocyanins in the juice and accounted for more than 51.5% of all anthocyanins, followed by peonidin-3-O-glucoside and malvidin-3-O-glucoside. In comparison with the juice, the placebo contained only minor amounts of anthocyanins.

### 3.2. Influence of Plasma Anthocyanins and Their Metabolites on HT-29 and Caco-2 Colon Cancer Cell Migration

**Figure 2:**
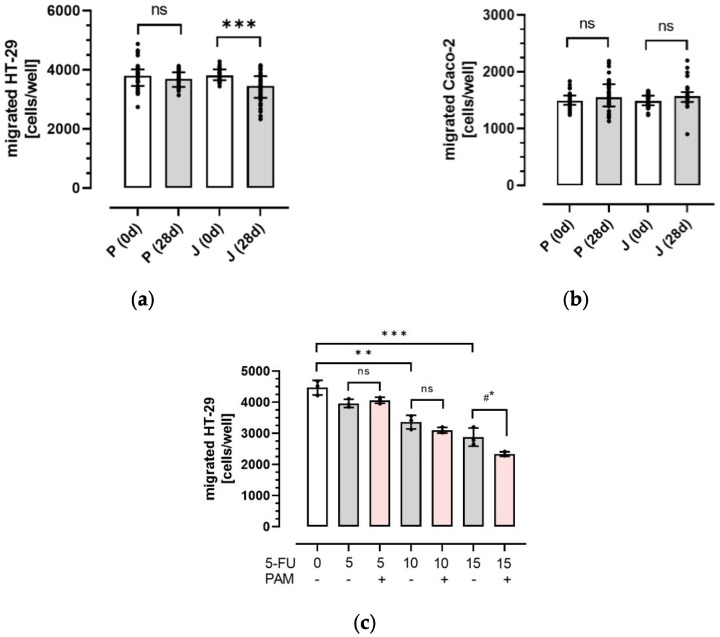
Migration of HT-29 and Caco-2 cells in vitro. HT-29 (**a**) and Caco-2 (**b**) cells were incubated with PAM from the anthocyanin-depleted placebo (P; *n* = 34) and anthocyanin-rich juice (J; *n* = 35) that were isolated before (0 d) and after 28-day (28 d) intervention. Migration was studied in a Boyden chamber with collagen-coated transwells. Basal cells in the lower chamber were measured after 36 h and migrated cell counts were detected fluorometrically as described in the Methods section. HT-29 cells (**c**) were exposed to indicated concentrations of 5-FU alone (5, 10, and 15 µM) and with pooled PAM (*n* = 10; most effective from (**a**)). Values are presented as aligned dot plots with median and interquartile range (25th–75th) (**a**,**b**) or means with standard deviation (**c**). Significant differences were calculated with a mixed model with multiple comparison test (Šídák’s) or ANOVA with multiple comparison test (Šídák’s). Values were different with ** *p* < 0.01 and *** *p* < 0.001 compared with the corresponding controls or with #* *p* < 0.05 compared with 5-FU (15 µM) alone. ns, non significant.

Anthocyanins and their metabolites were extracted by SPE from plasma samples before (0 d) and after the intervention (28 d) with juice or placebo. Afterwards, dried PAM were solved in cell culture media and applied to the two colon cancer cell lines HT-29 and Caco-2. As shown in Figure 2, cancer cell migration was differently affected by incubation with PAM from both beverages. In case of HT-29 cells, PAM from the juice significantly reduced cancer cell migration from 3806 (3646–4013) to 3453 (3052–3787) cells per cavity (*p* < 0.001). In comparison, no inhibition was observed after exposure to PAM from the placebo (Figure 2a). In contrast to HT-29 cells, migration of Caco-2 cells was neither influenced by PAM from the placebo nor by PAM from the juice (Figure 2b). Therefore, this study shows that physiological concentrations of PAM significantly impair the migratory potential of HT-29 cells, whereas migration of Caco-2 cells was not affected.

To examine the effect of 5-fluorouracil (5-FU) on HT-29 cell migration, various concentrations of 5-FU were applied. 5-FU is a common chemotherapeutic agent in colorectal cancer [49,50] and it is known that flavonoids can interact with chemotherapeutic agents [51]. However, possible interactions between anthocyanins and 5-FU are largely unknown. As shown in Figure 2c, HT-29 cell migration was concentration-dependently inhibited by 5-FU treatment. Compared with untreated control cells, incubation of HT-29 cells with the most effective 5-FU concentration (15 µM) significantly reduced colon cancer cell migration from 4472 ± 231 to 2882 ± 289 cells per cavity (*p* < 0.001). To further investigate, possible synergistic or even antagonistic effects of PAM on 5-FU treatment, HT-29 cells were co-incubated with 5-FU and pooled PAM from volunteers (*n* = 10) that most effectively inhibited HT-29 cancer cell migration. In comparison with 5-FU alone (15 µM), co-incubation with pooled PAM further decreased HT-29 cell migration from 2882 ± 289 to 2338 ± 71 cells per cavity (*p* < 0.05), respectively (Figure 2c). These results indicate that physiological concentrations of anthocyanins and their metabolites may promote the effects of classical chemotherapeutic agents like 5-FU.

### 3.3. Influence of Plasma Anthocyanins and Their Metabolites on HT-29 and Caco-2 Cell Viability

**Figure 3:**
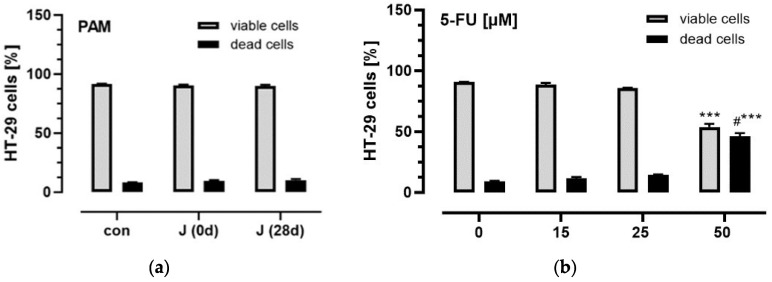
Effects of PAM (**a**) and 5-FU (**b**) on HT-29 cell viability. HT-29 cells were seeded at a density of 1 × 10^5^ cells/mL in 24-well plates in complete medium with or without PAM from the anthocyanin-rich juice that were isolated before (J (0 d)) or after 28-day intervention (J (28 d)) or with medium alone (con), as well as with different concentrations of 5-FU. After 36 h incubation, cells were washed twice with PBS, trypsinized and cell viability was measured using a Guava^®^ Muse^®^ Cell Analyzer. Data are expressed as bars [%] with standard deviation. Significant differences were calculated with ANOVA with multiple comparison test. Values were different with *** *p* < 0.001 compared with viable cells of the controls (con) or with #*** *p* < 0.001 compared with dead cells of the controls (*n* = 2).

To investigate possible cytotoxic effects of PAM on colon cancer cells, HT-29 were incubated with the pooled PAM from volunteers that most effectively inhibited HT-29 cancer cell migration (*n* = 10). As shown in Figure 3 viability of HT-29 cells was neither affected by incubation with PAM for 36 h, nor by 5-FU in low doses (≤25 µM). In contrast, 50 µM 5-FU significantly decreased viability of HT-29 cells compared with controls. In addition, PAM showed no cytotoxic effects in non-tumor cells (Figure S1).

### 3.4. Effects of the Anthocyanin-Rich Juice and the Anthocyanin-Depleted Placebo on Antioxidative Biomarkers in Plasma and Urine

**Figure 4:**
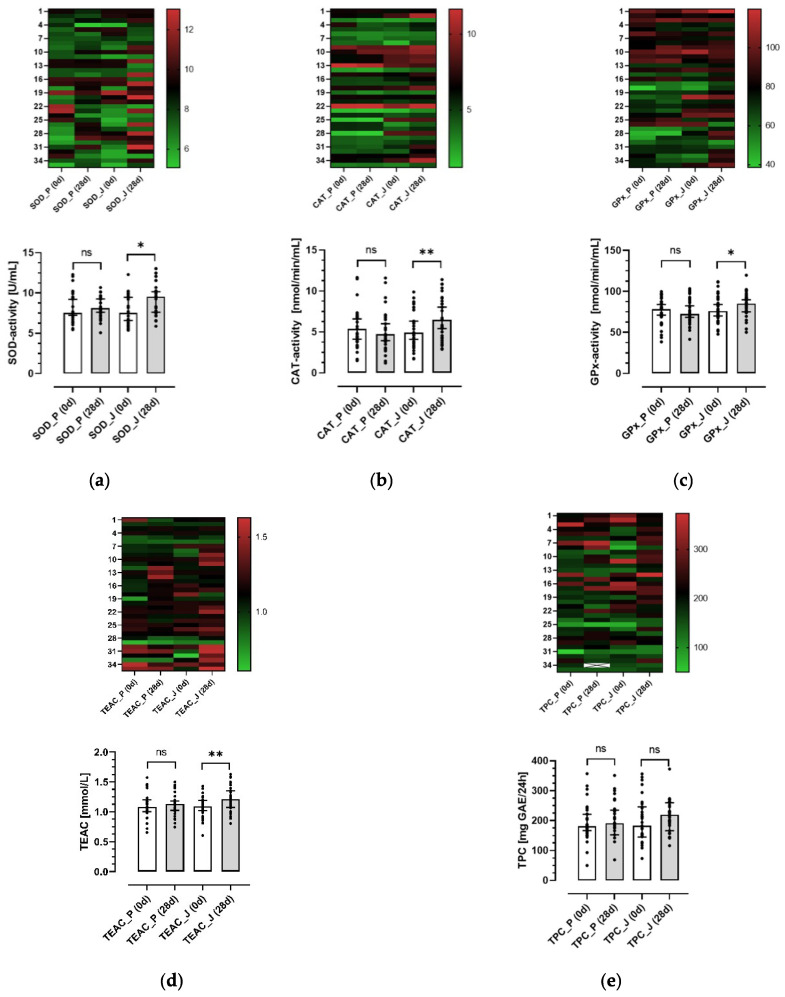
Effects of the anthocyanin-rich juice and the anthocyanin-depleted placebo on antioxidative parameters before and after intervention. Study participants consumed 0.33 L of the anthocyanin-depleted placebo (P) and anthocyanin-rich juice (J) over 28 days. Before (0 d) and after (28 d) intervention, blood samples were drawn and 24 h-urine samples were collected. Enzyme activities of SOD (U/mL) (**a**), CAT (nmol/min/mL) (**b**), GPx (nmol/min/mL) (**c**) as well as TEAC (mmol/mL) (**d**) were measured in plasma (*n* = 35). TPC (mg GAE/24 h) (**e**) was measured in urine (*n* = 34). Upper panel: Heatmap analyses showed single values for each participant. Lower panel: Values are presented as aligned dot blot bars with median and interquartile range (25th–75th). Significant differences were calculated with a mixed model with multiple comparison test (Šídák’s) or ANOVA with multiple comparison test (Šídák’s). Values after intervention were different with * *p* < 0.05, ** *p* < 0.005 to corresponding controls. ns, non significant.

Biomarkers of oxidative stress are significantly increased in CRC patients, whereas TAC is significantly lower compared with healthy controls [5,33]. Thus, we aimed to investigate whether long-term consumption of an anthocyanin-rich juice improves the antioxidant status of healthy volunteers. A significant increase of median (25th–75th percentile) plasma SOD activity from 7.53 (6.27–9.55) to 9.52 (7.21–10.69) U/mL (*p* < 0.001) was observed after a 28-day intervention with the juice. In contrast, no change in plasma SOD activity was found after consumption of the placebo (Figure 4a). Similarly, after ingestion of the anthocyanin-rich juice, plasma CAT and GPx activity significantly increased from 4.92 (3.77–8.00) to 6.42 (4.33–8.24) nmol/min/mL (*p* < 0.001) and from 75.92 (69.10–90.18) to 85.22 (74.28–91.96) nmol/min/mL (*p* < 0.05), respectively. Again, CAT and GPx activity remained unchanged after placebo intake (Figure 4b,c).

The antioxidant capacity of plasma samples was estimated as TEAC comprising the antioxidative capacity of both lipophilic and hydrophilic compounds. Comparable to antioxidative enzyme activities in plasma, the median TEAC value significantly increased after ingestion of the anthocyanin-rich juice from 1.09 (1.02–1.19) to 1.21 (1.07–1.35) mmol/mL (*p* < 0.001). However, after placebo intake no significant change was observed

(Figure 4d). In contrast to the observations for plasma antioxidative enzymes and capacity, no significant differences were found with respect to urine TPC after intake of both, placebo and juice (Figure 4e). Interestingly, heatmap analysis (Figure 4, upper panels) shows that GPx activity from vegetarians (volunteers 1–12) was higher than those of omnivores. In summary, these results show that consumption of an anthocyanin-rich juice for 28 days significantly improves the antioxidant status of healthy volunteers.

### 3.5. Correlation between Parameters of Antioxidant Capacity and Migration

**Figure 5:**
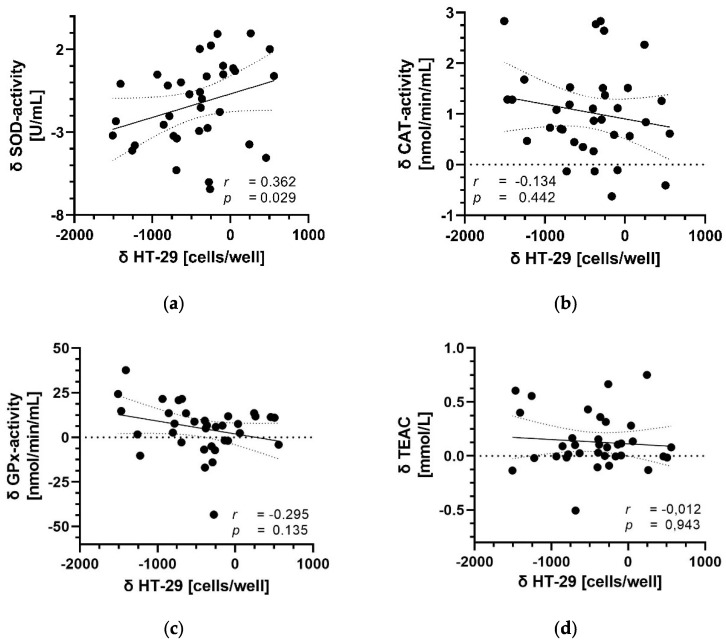
Scatter plots of antioxidant parameters and HT-29 migration. Correlation between HT-29 cell migration in vitro and SOD activity (**a**), CAT activity (**b**), GPx activity (**c**), and TEAC (**d**) in plasma. Correlation between the parameters were evaluated using Spearman correlation (r) with differences (δ) between values from day 28 and day 0 after anthocyanin-rich juice intake (*n* = 35).

We further aimed to investigate, whether the increase in antioxidant status parameters in the plasma of healthy volunteers after 28-day consumption of the anthocyanin-rich juice was correlated with the significant decrease of HT-29 colon cancer cell migration that was observed in vitro (Figure 5). In fact, the higher the increase in SOD activity in plasma after consumption of the anthocyanin-rich juice (Figure 5a), the higher the anti-invasive effect was on HT-29 cell migration in vitro (*p* < 0.05). In contrast, no correlation was observed between other antioxidant parameters in plasma, indicating a possible link between plasma SOD activity and colon cancer metastasis.

## 4. Discussion

Considering that about every fourth colon cancer patient displays distant metastases after resection [52] and cancer mortality is mainly due to metastasis lesions [52,53], searching for new therapeutic agents to suppress cancer cell invasion and migration is crucial. However, data from human studies regarding the effects of anthocyanins on colon cancer progression are scarce and to date, it has not yet been clarified whether anthocyanins, particularly at physiological concentrations, have an impact on cancer cell migration in vivo. To overcome these limitations, the present study was designed to determine the anti-migratory effects of anthocyanins and their plasma metabolites on cell migration after long-term ingestion of an anthocyanin-rich grape/bilberry juice. Therefore, we combined a placebo-controlled, double-blinded, cross-over intervention study with in vitro migration studies of two colon cancer cell lines, HT-29 and Caco-2. Healthy students daily consumed 0.33 L of an anthocyanin-rich juice in comparison to an anthocyanin-depleted placebo for 28 days. Blood samples were drawn before and after intervention. Subsequently, plasma samples were used to isolate PAM that were applied to a collagen-coated Boyden chamber for the measurement of colon cancer cell migration. To the best of our knowledge, this is the first study that showed that physiological concentrations of PAM significantly impair the migratory potential of HT-29 cells. In contrast, migration of Caco-2 cells was not affected. Although both cell lines were isolated from colon adenocarcinomas, they show differences in pheno- and genotypes, as well as in their grade of differentiation [54]. Hence, it is not surprising that undifferentiated HT-29 cells showed an obviously higher migratory potential compared with the more differentiated Caco-2 cells, which is underlined by the much higher number of migrated HT-29 cells in our experiments.

One of the most common and effective chemotherapeutic agents used for the treatment of CRC is 5-FU [50], although the benefit from 5-FU is often compromised by chemoresistance [49]. Therefore, it is vital to search for adjuvant agents and to investigate possible synergistic or even antagonistic effects of PAM on 5-FU treatment. Indeed, co-incubation of HT-29 cells with 5-FU and PAM further decreased cell migration compared with 5-FU treatment alone. Similarly, Li et al. recently reported that black raspberry anthocyanins significantly increased the anti-proliferative and anti-migratory effects of 5-FU and Celecoxib in colon cancer cell lines [55]. However, proliferation of Caco-2 cells was not affected by single treatment with black raspberry anthocyanins [55]. Accordingly, physiological concentrations of anthocyanins and their metabolites might possibly enhance the anti-migratory effect of 5-FU in Caco-2 cells. However, a possible synergistic effect of PAM and 5-FU from all volunteers was not investigated in the present study due to the limited sample material.

Metastasis is a multistep process that comprises cell migration from the primary tumor site, cell invasion, attachment to endothelial cells, extravasation, proliferation, and angiogenesis at the distal site [53,56]. Degradation of the extracellular matrix, especially collagen, mediated by proteolytic enzymes such as MMP-2 (gelatinase-A) and MMP-9 (gelatinase-B), is known to be the first step and plays a crucial role during cell migration and invasion [31,57]. While the anti-migratory and anti-invasive effects of anthocyanins have been reported for several cancer types, data regarding their impact on colon cancer cell migration are limited. The migration and invasion of HT-29 cells was significantly inhibited by an anthocyanin extract from *Vitis coignetiae Pulliat*, a member of the grape family, by suppressing MMP-2 and MMP-9 expression, which was likely due to the inhibition of NF-κB activation [25]. The inhibitory effects of anthocyanins on MMP-2 and MMP-9 expression were also shown in HCT-116 colon cancer cells [26]. Interestingly, Zhang et al., recently reported that black raspberry anthocyanins increased miR-24-1-5p expression in colon cancer cell lines, whereas miR-24-1-5p overexpression was associated with a significant decline of HCT-116 and Caco-2 migration [27]. However, these anti-invasive and anti-migratory effects were only shown at concentrations much higher than anthocyanin levels usually observed in plasma. Nevertheless, we have recently shown for the first time that physiological concentrations of anthocyanins and their metabolites isolated from the plasma of healthy volunteers are able to reduce tumor cell migration of the pancreatic cancer cell line PANC-1 in vitro [31]. This was accompanied by a significant reduction of ROS and decreased MMP-2 and MMP-9 levels, as well as NF-κB mRNA expression, suggesting that physiological concentrations of PAM may be adequate due to synergistic and additive effects. Although in the present study the underlying mechanisms remain unknown, it is most likely that inhibition of HT-29 cell migration is also attributed to suppression of the NF-κB pathway, as well as decreased expression of MMPs. In the present study the applied anthocyanin-rich juice was made from an eighty/twenty mixture of red grapes and bilberries with peonidin-3,5-O-diglucoside, malvidin-3,5-O-diglucoside, and peonidin-3-O-glucoside representing the major anthocyanins. It has been shown that peonidin-3-O-glucoside significantly inhibits MMP secretion and migration of lung cancer cells [58]. In addition, peonidin has been reported to be the most potent anthocyanidin comparing the anti-migratory and anti-invasive capabilities of the five anthocyanidins cyanidin, malvidin, peonidin, petunidin, and delphinidin [59]. Peonidin significantly suppressed migration and invasion of highly invasive H1299 cells by about 20% even at a relatively low concentration (6.25 µM) [59]. Furthermore, synergistic anti-migratory and anti-invasive effects have been reported for a mixture comprising the five aglycons at equimolar concentrations [59]. However, this approach did not consider the impact of anthocyanin metabolites, which are much more abundant in plasma compared with native anthocyanins [38]. In addition, the bioavailability of native anthocyanins is low due to (1) their low stability and degradation at physiological pH values in the small intestine, (2) their metabolization through Phase II enzymes, and (3) their metabolism by the intestinal microbiota [60,61,62]. Regarding the pharmacokinetics of anthocyanins, native anthocyanins reach maximum plasma levels 30–60 min after ingestion [38]. In contrast to the ANTHONIA study [21], blood samples were drawn approximately 6 h after juice ingestion in the present study. At this time, Phase-II as well as microbiota-derived metabolites are present in the systemic circulation [63]. Therefore, the present study suggests that not only native anthocyanins but also their metabolites exert potent anti-migratory properties. However, we did not identify which anthocyanins and metabolites are responsible for the observed anti-migratory effects. Therefore, characterization of these compounds should be addressed in future studies.

Epidemiological studies indicate protective effects of dietary polyphenols against oxidation- and inflammation-related diseases such as cancer, cardiovascular diseases, diabetes mellitus, and neurodegenerative diseases [64,65]. It has been shown that the TAC of CRC patients is significantly lower compared with healthy controls [5,33]. Furthermore, CRC progression and metastasis are also associated with a significant decline in TAC, as well as lower CAT and GPx activities in serum and plasma, respectively [5,66]. Therefore, CRC patients may be more vulnerable to oxidative stress and the administering of anthocyanins might improve the antioxidant capacity, overall health, and outcome of CRC patients. Our results show that daily intake of an anthocyanin-rich juice for 28 days significantly improves plasma antioxidant capacity of healthy volunteers determined by enzymatic and non-enzymatic biomarkers. Plasma antioxidant capacity, measured as TEAC, was significantly higher after consumption of the anthocyanin-rich juice, whereas no change in TEAC was seen after ingestion of the anthocyanin-depleted placebo. Similarly, activities of the antioxidant enzymes SOD, CAT, and GPx were significantly increased after the juice, whereas the enzyme activities were not affected by the placebo. These results are in line with our recent findings from the ANTHONIA study [21]. Interestingly, GPx activity was not affected after consumption of an anthocyanin-rich grape/bilberry juice for 14 days [21]. Discrepancies in the results for GPx activity may be explained by different intervention times. In addition, there were slight differences between the grape/bilberry juices during the two studies. Both juices were made from an eighty/twenty mixture of red grapes and bilberries. Whereas in the ANTHONIA study the two grape varieties, *Dakapo* and *Accent*, were used, only the variety *Accent* was utilized in the present study, resulting in the different anthocyanin pattern of the juices. Thus, malvidin-3-O-glucoside and peonidin-3-O-glucoside were the major anthocyanin in the ANTHONIA study, whereas peonidin-3,5-O-diglucoside and malvidin-3,5-O-diglucoside represent the main anthocyanins in the present study. However, expression of the antioxidant enzymes SOD, CAT, and GPx is controlled by the redox sensitive transcription factor nuclear factor-erythroid 2 p45-related factor 2 (Nrf2) [67]. It is well known that anthocyanins, but also their gut-derived metabolites, are able to activate the Nrf2 pathway and consequently upregulate the defense against ROS and oxidative stress [68,69]. Compared with cells from the primary tumor site, metastatic cancer cells displayed higher ROS concentrations [6]. The ROS lowering effects of anthocyanins and their degradation products (gallic acid, syringic acid, protocatechuic acid, and phloroglucinol aldehyde) have been demonstrated in different colon cancer cell lines [67]. In addition, we have recently shown that physiological plasma concentrations of PAM significantly reduce ROS generation in PANC-1 cells [31]. However, possible ROS lowering effects of PAM in HT-29 and Caco-2 cells have not been investigated in the present study. Recently, Yang et al., 2021 reported that blueberry anthocyanins significantly increased SOD activity in the lungs and livers of mice in vivo. In addition, in these organs reduced metastasis of breast cancer cells, which are known to be highly invasive, was observed [70], indicating a possible link between SOD activity and metastasis. However, to the best of our knowledge no study so far has investigated possible associations between the enzymatic and non-enzymatic antioxidant capacity in plasma and colon cancer cell migration. Interestingly, the higher the increase of SOD activity in plasma after 28 days intervention with the anthocyanin-rich juice, the higher the anti-invasive effect on HT-29 cell migration was in vitro. Although this correlation was weak, the impact of anthocyanins and their metabolites on tumor metastasis is much more complex in vivo. Besides direct effects on cancer cells, PAM may also influence other cell types such as immune cells [70] and consequently the microenvironment and migratory potential of tumor cells. To date, the role of anthocyanins during tumor migration of colon cancer patients is not fully understood and should be further investigated.

The present study has some limitations. First, participants were advised to adhere to a diet low in phenolics and anthocyanins during the run-in and intervention phase. These strong dietary limitations may not reflect a healthy and balanced diet. Furthermore, nutritional limitations were the reason for the high drop-out rate or termination of the study. Therefore, future studies should address whether the consumption of anthocyanin-rich beverages in addition to a normal diet also show health-promoting effects. Secondly, it remains to be shown which plasma anthocyanins and metabolites, as well as molecular mechanisms, are responsible for the anti-migratory effects. Finally, it could not be ruled out that the observed effects are in part mediated by other phenolic compounds also present in the anthocyanin-rich grape/bilberry juice.

## 5. Conclusions

To the best of our knowledge, we demonstrated for the first time that plasma-isolated anthocyanins and their metabolites significantly decrease migration of colon cancer cells in vitro. Furthermore, our data indicate that physiological concentrations of anthocyanins and their metabolites may enhance the efficacy of classical chemotherapeutic agents like 5-FU.

## Figures and Tables

**Table 1:** Anthocyanin composition of the anthocyanin-rich juice and the anthocyanin-depleted placebo ^1^.

	Anthocyanin-Rich Juice		Anthocyanin-Depleted Placebo	
Anthocyanins	(mg/L)	(%)	mg/L	(%)
peonidin-3,5-O-diglucoside	346 ± 12.5	36.8	1.7 ± 0.02	26.9
malvidin-3,5-O-diglucoside	138 ± 8.4	14.7	0.88 ± 0.06	14.0
peonidin-3-O-glucoside	83.5 ± 6.4	8.9	0.37 ± 0.01	5.9
malvidin-3-O-glucoside	63.4 ± 3.8	6.7	0.30 ± 0.01	4.7
delphinidin-3-O-glucoside	61.5 ± 2.9	6.5	0.80 ± 0.03	12.7
delphinidin-3-O-galactoside	53.6 ± 0.6	5.7	0.75 ± 0.01	11.9
delphinidin-3-O-arabinoside	53.4 ± 1.6	5.7	0.56 ± 0.02	7.4
petunidin-3-O-glucoside	43.7 ± 1.9	4.6	0.37 ± 0.02	5.8
cyanidin-3-O-arabinoside	27.2 ± 1.7	2.9	0.11 ± 0.02	1.7
cyanidin-3,5-O-diglucoside	18.2 ± 1.6	1.9	0.29 ± 0.00	4.6
malvidin-3-(6”-O-coumaryl)-5-O-diglucoside	17.1 ± 0.4	1.8	n.d.	n.d.
petunidin-3-O-galactoside	13.2 ± 0.3	1.4	0.11 ± 0.01	1.8
petunidin-3-O-arabinoside	8.8 ± 0.0	0.9	0.05 ± 0.01	0.8
malvidin-3-O-arabinoside	5.3 ± 0.0	0.6	0.02 ± 0.00	0.3
peonidin-3-O-galactoside	4.3 ± 0.0	0.5	0.01 ± 0.00	0.1
delphinidin-3,5-O-diglucoside	3.4 ± 0.0	0.4	0.09 ± 0.01	1.4
Sum	942 ± 10	100	6.3 ± 0.5	100

^1^ Juices were analyzed by LC–MS (*n* ≥ 2) and data are expressed as mean ± SD mg cyanidin-3-O-glucoside equivalents per L.n.d., non-detectable

## Data Availability

The data are not publicly available due to European data protection regulations.

## Supplementary Materials

The following supporting information can be downloaded at: https://www.mdpi.com/article/10.3390/antiox11071341/s1, Table S1: Foodstuffs that were restricted or not allowed during the run-in and 28-day intervention period; Table S2: Baseline characteristics and mean dietary intake of the study population during the two intervention periods (*n* = 34); Figure S1: Effects of PAM on HUVE cell viability.
