# Supplementary material for: Influence of Plasma-Isolated Anthocyanins and Their Metabolites on Cancer Cell Migration (HT-29 and Caco-2) In Vitro: Results of the ATTACH Study
Source: Antioxidants (Basel). 2022 Jul 8;11(7):1341. doi: 10.3390/antiox11071341 (PMC9311669; doi:10.3390/antiox11071341)
Supplement: Supplementary file 1 [file antioxidants-11-01341-s001.zip › antioxidants-1773870-supplementary.pdf]

Supplementary Material

Influence of plasma isolated anthocyanins and their metabolites on cancer cell migration (HT-29 and Caco-2): Results of the ATTACH study

Authors:

Inken Behrendt [1*], Isabella Röder [2], Frank Will [2], Hamza Mostafa [3,4], Raúl Gonzalez-Dominguez [3,4], Tomás Meroño [3,4], Cristina Andres-Lacueva [3,4], Mathias Fasshauer [1], Silvia Rudloff [5] and Sabine Kuntz [1]

[1] Department of Nutritional Science, Human Nutrition, Justus-Liebig-University, Giessen (Germany)

[2] Department of Beverage Research, Hochschule Geisenheim University (Germany)

[3] Biomarkers and Nutrimetabolomics Laboratory, Department of Nutrition, Food Sciences and Gastronomy, Food Innovation Network (XIA), Nutrition and Food Safety Research Institute (INSA), Facultat de Farmàcia i Ciències de l'Alimentació , Universitat de Barcelona (UB), Barcelona (Spain)

[4] Centro de Investigación Biomédica en Red de Fragilidad y Envejecimiento Saludable (CIBERFES), Instituto de Salud Carlos III, Madrid, 28029 (Spain).

[5] Department of Nutritional Science and Department of Pediatrics, Justus-Liebig-University, Giessen (Germany)

* Correspondence: inken.behrendt@ernaehrung.uni-giessen.de

Table S1. Foodstuffs that were restricted or not allowed during the run-in and 28-day intervention period.

Foodstuffs allowed with **minor restrictions**[1]	Foodstuffs **not allowed**
**Milk and milk products**: milk, yoghurt and other milk products with fruit, nuts (no red fruit).<br><br>**Vegetables**: yellow and green paprika, leek, spinach, cauliflower, broccoli, onions (no red onions).<br><br>**Fruit**: bananas, pears, white grapes, apples (max. 1 piece/day), peaches, apricots (max. 1 piece/day), kiwis, pineapple.<br><br>**Breakfast cereals**: cereals (no wholemeal/ without red fruits/ dark chocolate/ bran).<br><br>**Fat, spices**: vegetable fat and oil, paprika spice, chili spice, cayenne pepper, curry spice.<br><br>**Sweets:** ice cream with fruits, nuts (without red fruit / dark chocolate), marmalade/ jam/ jelly (without red fruit).<br><br>**Allowed (per day):** 1 cup of tea, 1 cup of coffee, 1 glass of ale or glass of white wine (no red wine).<br><br>Please note: fruit and vegetable, if possible, have to be peeled before consumption, especially, if consumed more portions a day, forgo red fruits, no consume external leaves of salad, no consume cereal products, listed spice and mixed spices use sparing.	**Fruit**: blueberries, blackberries, cranberries, strawberries, bilberries, raspberries, currants, lingonberries, cherries, red grapes, pomegranate, plums, watermelon.<br><br>**Fruit containing foodstuffs:** red marmalade/ jellies, cake with red fruits, yoghurt with red fruits, ice cream with red fruits, desserts, muesli, muesli bars, cereals with wholemeal, dark chocolate, cocoa powder, gumdrop with fruit.<br><br>**Vegetables**: purple potatoes, purple carrots, tomatoes, red and orange peppers, red cabbage, beetroot, radicchio, red radish, rhubarb, red and black pulses (kidney beans, red lentils), eggplant, pumpkin, red onions.<br><br>**Other:** tomato sauce, puree, or paste, ketchup, red pesto.<br><br>**Avoid** red wine, fruit and vegetable juice, fruit spritzer, smoothies, fruit tea, green tea, coffee, rooibos tea, alcoholic drinks (bear, wine, schnapps, and liqueur).<br><br>Please note: anthocyanins may be food additives listed as number E 163.

[1]Foodstuffs were categorized according to their anthocyanin content based on data using the USDA database for the Flavonoid Content of Selected Foods (Release 3.3 (2018); http://www.ars.usda.gov/) or the database on polyphenol contents in food (Polyphenol-Explorer, Release 3.0 (http://www.phenol-explorer.eu/compounds)

Table S2. Baseline characteristics and mean dietary intake of the study population during the two intervention periods (n=34).

Dietary intake per day[1]	Intervention period 1	Intervention period 2
**Age,** *years*	24.4 ± 2.3	
**Weight,** *kg*	64.3 ± 11.7	63.2 ± 12.07
**BMI,** *kg/m²*	21.7 ± 2.6	21.3 ± 2.2
**Dietary intake (3-day protocol)**		
**Energy,** *kcal/d*	1698 ± 552	1855 ± 578
**Fat,** *g/d*	63.4 ± 17.9	64.4 ± 18.7
**Carbohydrates,** *g/d*	250.9 ± 63.2	245.8 ± 48.1
**Dietary fibre,** *g/d*	20.3 ± 5.3	19.4 ± 6.0
**Protein,** *g/d*	60.6 ± 16.4	54.7 ± 13.7
**Retinol equivalents,** *µg/d*	714 ± 263	682 ± 207
**ß-carotene,** *µg/d*	1,698 ± 1,520	1,387 ± 1,147
**Vitamin E,** *µg/d*	6,925 ± 6,630	7,201 ± 6,921
**Vitamin C,** *mg/d*	57.14 ± 34.2	63.88 ± 40.4

[1]Participants recorded their dietary intake over 3 days during each intervention period. Dietary records were analyzed using the DGE-PC professional software (Version 1.10.0.0) and values are expressed as means ± SD .

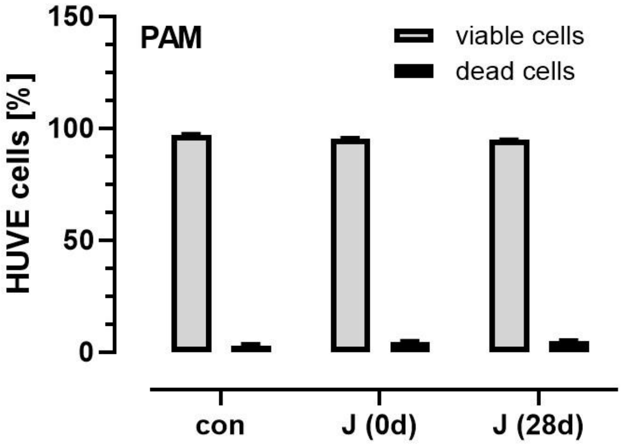

Figure S1. Effects of PAM on HUVE cell viability. Cells were seeded at a density of $1x10^5$ cells/mL in 24-well plates in complete medium with or without PAM from the anthocyanin-rich juice, which were isolated before (J (0d)) or after 28-days intervention (J (28d)) or with medium alone (con). After 36 h incubation, cells were washed twice with PBS, trypsinized and cell viability was measured using a Guava® Muse® Cell Analyzer. Data are expressed as bars [%] with standard deviation. Significant differences were calculated with ANOVA with multiple comparison test (n=2).
